# Comparing Plasticity of Response to Perceived Risk in the Textbook Example of Convergent Evolution of Desert Rodents and Their Predators; a Manipulative Study Employing the Landscape of Fear

**DOI:** 10.3389/fnbeh.2019.00058

**Published:** 2019-03-22

**Authors:** Sonny S. Bleicher, Burt P. Kotler, Joel S. Brown

**Affiliations:** ^1^Department of Environmental Science and Policy, George Mason University, Fairfax, VA, United States; ^2^Mitrani Department for Dryland Ecology, Blaustein Institutes for Desert Research, Ben Gurion University of the Negev, Beer-Sheva, Israel; ^3^Department of Biological Science, University of Illinois, Chicago, Chicago, IL, United States; ^4^Konevesi Research Station, Jyväskylän Yliopisto, Jyväskylä, Finland; ^5^Department of Integrated Mathematical Oncology, Moffitt Cancer Research Center, Tampa, FL, United States

**Keywords:** community ecology, common garden experiments, convergent evolution, ecology of fear, evolutionary game theory, habitat selection, predator-prey interactions, spatial ecology

## Abstract

Foragers process information they gain from their surroundings to assess the risk from predators and balance it with the resources in their environment. Measuring these perceived risks from the perspective of the forager can produce a heatmap or their “fear” in the environments, a so-called “landscape of fear” (LOF). In an intercontinental comparison of rodents from the Mojave and Negev Deserts, we set to compare families that are used regularly as examples of convergent evolution, heteromyid and gerbilline respectively. Using a LOF spatial-analysis on data collected from common garden experiments in a semi-natural arena we asked: (1) do all four species understand the risk similarly in the exact same physical environment; (2) does relative relation between species affect the way species draw their LOFs, or does the evolutionary niche of a species have a greater impact on its LOF?; and (3) does predator facilitation between vipers and barn owls cause similar changes to the shape of the measured LOFs. For stronger comparative power we mapped the LOF of the rodents under two levels of risk: low risk (snakes only) and high risk (snakes and barn owls). We found concordance in the way all four species assessed risk in the arena. However, the patterns observed in the LOFs of each rodent family were different, and the way the topographic shape of the LOF changed when owls were introduced varied by species. Specifically, gerbils were more sensitive to owl-related risk than snakes and at the opposite correct for heteromyids. Our findings suggest that the community and environment in which a species evolved has a strong impact on the strategies said animals employ. We also conclude, that given the homogenous landscape we provide in our arena and the non- homogenous patterns of LOF maps, risk assessment can be independent of the physical conditions under which the animals find themselves.

## Introduction

Colloquially fear is defined as a psychological emotion that drives anti-predator responses of an individual animal to risk related information it collects from its environment (Laundré et al., 2010; Clinchy et al., 2013). However—ecologically fear should be defined as the strategic response of an individual animal to risk related information it collects from its environment. This definition stands in contrast with the vague term of psychological emotion. Mathematically this translates to the solution of a game theoretic model balancing the tradeoffs of resources, energetic and reproductive, and safety (Brown et al., 1999; Bleicher, 2017). Those strategic responses, when measured on the physical landscape can provide a visualization of the way a population of individuals perceives the risk in the landscape in form of a topographic (or heat) map, a landscape of fear (LOF; Laundré et al., 2001, 2010). In this article, we use LOFs to observe how similar species from two desert communities respond to the risk from the same set of predators, and most importantly in the exact same physical environment. The selective pressures imposed by historical predators over evolutionary time manifest themselves in contemporary populations as strategic decisions and behavioral patterns in response to a community of modern predators, both known and novel (invasive). Thus, employing the LOF framework in such a comparison permits an investigation of how the lethality of historical predators has impacted the current space-use and decision-making of species.

Predators affect plant communities both directly by consuming herbivores and indirectly through behavioral effects on their prey (Ale and Whelan, 2008; Orrock et al., 2008; Sih et al., 2010). Perhaps the best-known example comes from the reintroduction of wolves to Yellowstone National Park. The risk from wolves frightens the elk, especially females, causing them to forage in less-risky habitat away from rivers. This permits the survival and renewed growth of willows and aspens (Ripple and Beschta, 2006). In turn, these stabilize the stream banks (Ripple and Beschta, 2006, 2012; Beschta and Ripple, 2009; Eisenberg et al., 2014). Using this example, Laundré et al. (2001) proposed the LOF as a framework for understanding the consequences of behavioral trophic cascades in landscapes that are spatially heterogeneous in risk of predation. In a recent review, Bleicher (2017) suggests the LOF can be used to metaphorically understand the animal’s *umwelt* (see Uexküll, 1909), the way it understands its environment. While physical aspects of a landscape may alter the cognitive responses of an individual animal to its surroundings, as in the example of waterways in Laundrè’s seminal article (2001), the LOF should be studied as an attribute of a population which can but will not always, be independent of these features.

We define the LOF as a “map,” a visualization, of how animals perceived the predation cost of foraging within the constraints of time and space. When an animal navigates the landscape in search of forage it balances the perceived risk through a number of energetically costly behaviors: vigilance, heightened senses, and even missed opportunities when the risk is deemed too high (Brown, 1999). The LOF, reflects the fundamental strategic-decisions an animal makes in its environment, habitat-use, and foraging behaviors. Thus, it is not surprising that the tool favored to measure the LOF has been the Giving-up Density (GUD) in over 60% of all manuscripts that chart the forager’s LOF (Bleicher, 2017). The GUD, a model describing the quitting harvest rates of foragers as a function of foraging and predation costs (Brown, 1988) is now used as a tool. The optimal patch-use model, as it was known originally, states that given diminishing-returns of resources in a patch, the amount of food left behind at a location by a forager would equal the inflection point where the costs related to foraging outweigh the energetic benefits (Bedoya-Perez et al., 2013).

**Figure 1:**
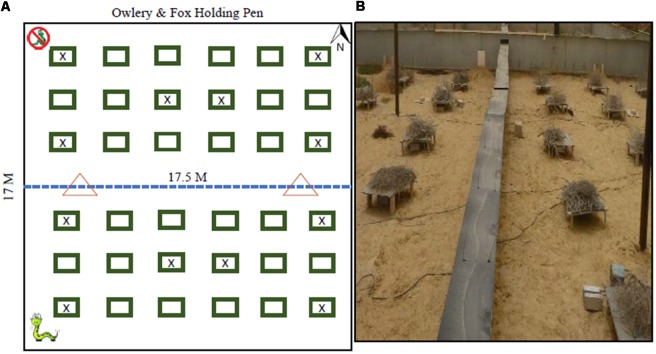
Vivarium Layout **(A)** where the north (top) has no snakes and the bottom has two snakes, one Sharan Horned Viper and on Sidewinder Rattle snakes corresponding to the snakes with whom each of the rodent families evolved. The rectangles represent trellises replicating bush microhabitat. Trellises with an (x) did not have a foraging patch. The dotted line is a see-through hardware cloth fence reaching 1.5 m above ground and the same depth below ground to prevent tunneling. The triangles are the location of “S”-shaped gates that restrict the movement of snakes but allow the ideal free distribution of rodents across the entire arena. **(B)** A picture of the setup.

We present here a large biogeographical comparison of convergent species from different genera to test whether physical and morphological convergence leads to behavioral convergence in those species, specifically in their LOF and the way these change under variations in predation risk within a semi-natural arena (located in Sde-Boker, Israel). Similarly, we also use this experiment to test whether imperfect behavioral-convergence (see Bleicher et al., 2018a), will result in an imperfect convergence of the LOFs. Our experimental arena, vivarium, is a 17 × 17 m aviary that was constructed to facilitate common-garden experiments with predators and prey populations. This setup allows for the measurement of animal foraging in a non-invasive, near-natural environment while achieving full control of predation-risk variables and removing inter-specific competition (Figure 1).

Our example compares two well-studied families that exhibit many ecological, behavioral, and physical similarities—desert rodents from the family Heteromyidae mostly from North America and the subfamily gerbillinae from Africa and Asia. We address, compare, and contrast the behavior of these rodents using habitat selection, patch use, and in a broader sense, foraging dynamics. In general, a forager’s LOF will be jointly influenced by its own species-specific aptitudes for detecting and evading predators, the structure of the physical environment insofar as particular features may favor the prey or the predator, and finally the perceived abundance and type of predators currently threatening the forager (Laundré et al., 2010; Bleicher, 2017).

While the intercontinental comparison is perhaps the most attention-drawing comparison this article provides, it is not our only objective. Within each family, we specifically chose one smaller species (20–35 g) and one larger (38–40 g) to determine whether body size differentially affects how rodents balance perceived risk and competition with other species. In both rodent families, the larger species is considered a stronger competitor (Thompson, 1982; Kotler et al., 1993b). There is strong evidence for anti-predator adaptations playing a key role in the mechanism of coexistence of these competing species (Kotler, 1984; Kotler and Brown, 1999). Additionally, series of manipulative experiments distilled the major environmental elements that allow for these competitors to coexist: most prominent among them being microhabitat partitioning between bush and open microhabitats (Rosenzweig, 1973; Kotler et al., 1993a); foraging substrate (Kotler et al., 2001); moonlight (Bouskila, 2001; Kotler et al., 2010), and temporal partitioning (Kotler et al., 1993b). To maximize our comparative ability, we brought our study populations into the same seminatural-arena and into a controlled and relatively featureless environment in which we controlled foraging resources, population size, and risky habitat (offering foraging patches in open and bush microhabitats).

This set-up allows us to ask three questions using the comparison between these four rodent’s LOFs:

(1) do all species of rodents exhibit similar LOFs across the various predation conditions?

If the rodents “understand” the risk in the same ways, areas ranked as high or low risk would be similar among the species. We expect the larger rodents to have similar patterns and the smaller competitors to have similar ones. This expectation leads to the second question.

(2) are similarities in the LOFs most striking for rodents of similar body sizes regardless of continent of origin or for rodents originating from the same desert even when they differ in body size?

We aim to separate the role of historical (evolutionary) risk of predation, the ghost of predator past, from the relative competitive ability of a species in shaping a population’s LOF. If the two heteromyids respond in a similar way and the two gerbils respond in a different, but mutually similar way, it would undermine our assumption of evolutionary convergence between the rodent families, at least in their behavior.

(3) do owls cause changes only in the elevation of the LOF or in its shape as well?

This query is aimed to question the way these species assess risk; how they understand predation risk as a whole. A LOF that rises and falls based on the cumulative risk posed by predators, but maintains its shape (key topographic features), suggests that historical (evolutionary-scale) predation risk had a strong impact in the species’ spatial distribution, i.e., the LOF reflects a fixed pattern that is tweaked (or turned on and off) based on the level of risk in the environment.

In contrast, a species could show plasticity in response to cumulative risk posed by predators. A species that redraws its LOF based on a focal feature of risk is most likely a product of evolution in a system where different predators applied different predatory pressures, and possibly at different rates of interaction. In such a situation, the cost-benefit analysis governing the foraging behavior of that species would focus on the greatest risk in the environment before factoring in lesser costs.

## Materials and Methods

### Site

We conducted our experiments in a semi-natural, outdoor enclosure (vivarium 17 × 17 m) located on the Sede Boker Campus of Ben-Gurion University, Blaustein Institutes for Desert Research, Midreshet Ben-Gurion, Israel (N 30° 51’ 25.978”, E 34° 46’ 51.284”). The vivarium was divided into quadrants where the southern half of the arena housed two vipers and the northern was a snake-free control (Figure 1). The vipers’ movement was restricted by gates that permitted only the rodents to move freely between quadrants. The vivarium, covered with a wire net 7.5 m high, forms and aviary that allowed for barn owls to fly freely within the structure. The owls were housed and released, one at a time, from cages adjacent to the facility. To minimize actual depredation of rodents, the owls were fed prior to release and returned to their boxes at sunrise.

### Species

We brought together one large and one small coexisting desert rodents from each continent, two common gerbils from the Negev Desert of Israel and a kangaroo rat and a pocket mouse from the deserts of the southwestern United States, to a common and controlled setting in the Negev Desert. The Negev Desert gerbils include the greater Egyptian gerbil *Gerbillus pyramidum* (GP), 40 g, and Allenby’s gerbil *Gerbillus andersoni allenbyi* (GA), 30 g (Goodfriend et al., 1991). The North American Desert rodents include Merriam’s kangaroo rat *Dipodomys merriami* (DM), 45 g (Lancaster, 2000), and the desert pocket mouse *Chaetodipus penicillatus* (CP), 22 g (Chebes, 2002). All are nocturnal desert granivores commonly found on sandy substrates such as sand dunes. All four rodents have adaptations to reduce the risk of predation, including saltatorial locomotion for enhanced escape abilities and auditory adaptations to increase hearing acuity. These adaptations are especially well developed in the kangaroo rats (Kotler, 1984; Randall, 1997).

We brought wild-caught vipers, trapped at locations where they would come in contact with wild populations of the above-mentioned rodents, to the same facility. We caught sidewinder rattlesnakes *(Crotalus cerastes)*, 35–60 cm mean length, from the Mojave Desert (Webber et al., 2016) and Saharan horned vipers (*Cerastes cerastes*), 30–60 cm mean length, from the Negev Desert (Anderson, 2011). Both snakes side-wind, burrow in the sand (usually under bushes) and feed on a variety of rodents and lizards (Ori, 2000; Anderson, 2011).

The animal collection was done respectively in the Mojave and Negev Deserts. The heteromyids were trapped between April-June 2012 predominantly in the Parker Dunes area (N 34°9’7.969”, W 114°7’34.245”) and supplemented by individuals from the San Bernardino (AZ) area (N 31°23’22.082”, W 109°11’ 22.851”). The sidewinders were collected in the Avra Valley alongside country roads (N 32°24’49.335”, W 111°29’38.138”) in two collection bouts in the spring of 2011, and 2012. The gerbils in Israel were collected in the Mashabim Dunes (N 31°0’14.531”, E 34°44’47.31”) and the horned vipers on the border between Israel and Egypt at Be’er Milka (N 30°57’4.609”, E 34°23’10.821”). The gerbils were trapped in the spring of 2011 (GA) and 2012 (GP). The experiments were conducted within a year of trapping the animals to reduce the habituation to lab conditions. All the North American species were held in quarantine for 4 weeks prior to exportation to Israel. When in Israel, all the animals were held in climate-controlled animal-rooms at the Blaustein Institutes for Desert Research within 300 m from the experimental vivarium. The rodents we used in these experiments were all male except for GPs where the population had 60% males (due to a shortage of males caught from the wild). We used only males in this experiment to comply with importation regulation for the Heteromyid rodents limiting the risk of releasing a possible invasive species (see Long, 2003).

### Animal-Care

When not in experiments, the animals were housed in climate and light controlled animal husbandry rooms at the university. Rodents were caged in standard individual rodent cages lined with sterilized sand. They were fed nightly 3 g of millet, and the sand was replaced every 2-weeks according to animal care protocol. The animals were fed weekly with a handful of clover to sustain their water intake. Snakes were held in locked storage-bins 80 × 40 × 40 cm lined with sand and were given water (changed every few days) and a reptile heat lamp. Each snake was fed one live feeder mouse (*Mus musculus*) per week and their bins cleaned every 2 weeks.

### Experimental Set-Up

#### Dates and Animals

The measurements were collected for each species in the absence of direct competition. This allowed us to make a comparative study of the effects of predation risk in the exact same setup in the absence of competition stressors. To allow for equal measurement of resource-use, we populated the vivarium with roughly the same biomass of rodents corrected for metabolic rate, leading to: 24 Allenby’s gerbils from June-August of 2011, 16 Greater Egyptian gerbils from June-July of 2013, 16 Merriam’s kangaroo rats from July-August of 2012, and 24 desert pocket mice from September-October of 2012. We ran each experiment for two lunar months. We measured the perception of risk each individual rodent perceived from the snakes in the experiment before entering the experiment and once it was taken out of the experiment (Bleicher et al., 2018a).

Based on RFID pit tags, implanted subcutaneously in each rodent, we tracked depredation in the vivarium. We found (in owl pellets, snake fecal matter and exit inventory) that a total of seven GA, five CP, three DM, and nine GP were depredated or died of natural causes during the experiment. During the experiment, for every pit tag found in an owl pellet, we released another individual in roughly the same location where the depredated individual was last observed (data from RFID antennae).

#### The Environment

Our vivarium mimics a dune habitat with a layer of 10 cm of sand covering a loess-clay subflooring with escape fencing buried to 1.5 m below ground. To replicate the sparse vegetation cover in semi-stabilized desert dunes we artificially created heterogeneity by adding 36 bramble-covered trellises in a grid of 6 × 6 each situated 2 m from the next trellis. Each trellis (80 × 50 × 15 cm) at each station was topped with a pile of cut brush to create a spherical “bush” approximately 1 m in diameter (Figure 1B). The environment beneath each trellis provides a sheltered environment mimicking a bush microhabitat, while the space between trellises replicates the open space naturally occurring between vegetation clumps in the deserts where our animals were trapped.

The vivarium was divided into two quadrants with a hardware-cloth fence (1 m above ground and 1.5 m underground) that separated the northern and southern halves. On this divider, we installed two rodent gates, 8 m apart, that allowed rodents to pass freely from section to section. This measure allowed the rodents to distribute themselves according to an ideal free distribution. These gates were engineered to allow the movement of the rodents and restrict the movement of snakes. We avoided measuring the foraging of the rodents at the four corners and central two trellises of each quadrant. In addition, the distribution of these stations purposefully minimized and equalized the distance of patches from an edge of the enclosure keeping each station 3 m away from a fence or another station (Figure 1A).

#### Data Collection

We measured the LOF using a grid of 24 foraging patches sieved nightly to obtain a GUD measurement. Each patch (38 × 28 × 8 cm) held 3 liters of sand and was stocked each evening with 3 g of millet. At sunrise, each patch was sieved and weighed to 1/100 g and logged for analysis. Unforaged patches were collected and reset daily as well.

Every month, we ran two eight-night rounds. Each round comprised of four nights with owls and four nights without owls. Each month, one round was centered on the full moon and the other the new moon, for a total of 16 data collection nights per month and 32 nights per species.

We set the patches in rows of four with two under trellises recreating a bush microhabitat, and two placed adjacent (10 cm away) to two additional trellises representing the open microhabitat. Every 2 weeks we altered the patches’ microhabitat at each of the 24 stations.

We have to state a major caveat for the experiment with the Allenby’s gerbils (smaller Negev species). In that experiment, which was run first and acted as a pilot, we tested two additional layers of complexity not tested in the other species. We ran four six-night rounds per month centered around each of the four moonphases (new, waxing, full, waning), with only two nights per moonphase with the owls (Bleicher, 2014; Bleicher et al., 2016). Additionally, the experiment also added rotations with a muzzled red fox for two nights per moonphase. Based on these differences we ended up using a small subset of the data for this analysis and loosing statistical power from eight nights in other species to two per round in these smaller gerbils.

### Data Analyses

#### General Effects

We ran a random-forest decision tree analysis in Statsoft Statistica. This analysis, best described as a categorical principal component analysis, uses a Bayesian approach to produce the likely major effects (splits) the data can produce organized by likelihood from most likely and important to the less robust effects at the final nodes. In this analysis, we input GUD as our independent variable, and species, microhabitat, snakes and owls as our dependent variables.

#### Station Effect

To account for station effects within quadrants, we used a general linearized model with GUD at a particular station as the dependent variable and rodent species, owls, quadrants, days (nested within owls), and station (nested within quadrants) as the independent variables. The objective of this analysis was to: (1) verify a station effect after accounting for quadrant or aviary-wide effects of the owl and snakes; and (2) determine the main effects of owls to see how they would influence the average “elevation” of the rodents’ LOF. A significant station-effect suggests that the variation in perceived risk is spatially dependent as opposed to being explained wholly by the categorical division of risk treatments.

While the station effects may be significant as a whole for all rodent species, we also wanted to compare whether there was a fixed pattern to risk distribution each species associated with each station. Given an expected strong effect to presence and absence of snakes we ranked the level of fear in each of the 12 stations in each quadrant based on mean GUDs (combining owl treatments, microhabitat, moonphase, and the whole 2 months of data collection). The highest GUD, highest risk, was given a rank of 12 and the lowest GUD, safest station, given a rank of 1. We ran a Freidman’s test of concordance for each quadrant with species as blocks (*n* = 4) and stations as treatments (*k* = 12). A significant concordance would suggest that the spatial distribution of risk in the arena was not random and that the rodents assessed the physical attributes outside of the experimental manipulations similarly (e.g., walls, fences, structural predator holding pens).

#### Landscape Shape

We wanted to examine the effect of changing risk on low-risk nights compared to the risk on high-risk nights. To do so, we regressed the GUD at each station on nights when owls were present in contrast with nights when owls were absent. A strong correlation would suggest that the LOF does not change qualitatively, but the features remain the same, and an increase in risk “elevation,” i.e., a higher mean GUD, would reflect increased risk (see Laundré et al., 2010). Low correlation and flat slopes would suggest that the landscape features are redrawn based on the changed predator community.

#### LOF Maps

A visualization of the data was run by creating raster-maps using a smoothing function of distance, weighted least squares (DWLS) with the default tension of 0.5 (see Iribarren and Kotler, 2012). A map was created for each combination of owl treatments by rodent species for a total of eight maps.

## Results

**Figure 2:**
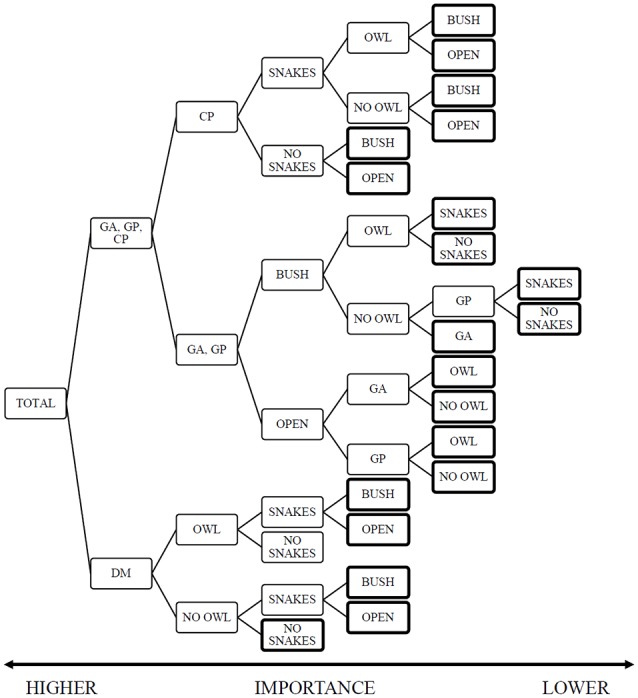
Random-Forest Decision-Tree with Giving-up Density (GUD) as the dependent variable and species, snake-treatment owl and microhabitat as the independent variables. The figure is read from left to right with greater value to the initial nodes (left) than to final nodes marked with a bold outline. This analysis highlights the difference between the rodent families, where gerbils rank microhabitat as a greater importance than heteromyids. The heteromyids diverge in their focal predator, kangaroo rats ranking owls as a greater contributing factor to their foraging and pocket mice responding to snakes first. The data structure for this tree (Supplementary Table S1) provides the sample size, mean GUD and variance for each node).

The random forest analysis produced a model with training and test risk estimates of 0.47 ± 0.02 and 0.52 ± 0.3 respectively. The decision tree highlights the importance of the rodent species (importance rank 1.0), followed by the presence of owls [0.1], and both the microhabitat and snakes at the lower end [0.07]. The model offers a clear split between the importance of variables in the foraging decisions of heteromyids and gerbils (Figure 2, Supplementary Material, Appendix I). The heteromyids rank the predators, both snakes and owls as greater importance than habitat heterogeneity in the form of microhabitat. On the other hand, gerbils are sensitive to landscape variations and rank snakes as the lowest impact.

The general linear model (*N* = 2,688, *R*^2^ = 0.537) found that for each species, GUDs differed significantly among stations (Table 1). All species combined responded with preferences for the bush over the open microhabitat, preferred the control over the quadrant with snakes, and foraged more on nights without owl presence. The model also found a significant difference among species’ foraging tenacity and for each species an interaction of owl presence with microhabitat and owl presence with snake treatment (quadrant). We do not offer in-depth examination here of these differences as they distract from the main purpose of this article, and are the basis for a number of articles published separately (Bleicher, 2014; Bleicher et al., 2016, 2018a; Kotler et al., 2016).

**Table 1:** ANOVA comparing Giving-up Densities (GUDs) as testing for a station effect and effects of owl presence snake treatments and microhabitat on patch use combined for all four rodents (*N* = 2, 688, *R*^2^ = 0.537).

Source	Type III SS	*df*	MS	*F*-Ratio	*p*-Value
LONGITUDE (X)	0.224	1	0.224	0.442	0.506
LATITUDE (Y)	1.886	1	1.886	3.732	0.053
Y × X	2.614	1	2.614	5.173	0.023
SPECIES × X × Y	22.717	3	7.572	14.983	0.000
OWL	68.992	1	68.992	136.506	0.000
MICROHABITAT	20.355	1	20.355	40.273	0.000
SPECIES	537.083	3	179.028	354.221	0.000
SNAKE	9.392	1	9.392	18.583	0.000
MICROHABITAT × OWL	0.567	1	0.567	1.122	0.290
SPECIES × OWL	5.195	3	1.732	3.426	0.016
SPECIES × MICROHABITAT × OWL	4.912	3	1.637	3.239	0.021
SNAKE × SPECIES × OWL	4.965	3	1.655	3.275	0.020
SNAKE × SPECIES × MICROHABITAT	2.453	3	0.818	1.618	0.183
Error	1,345.409	2,662	0.505		

*Abbreviations: SS, sum of squares; df, degrees of freedom; MS, mean squares; p-Value, Probability of Type I error; owl, barn owl presence; species, rodent species; snakes, snake treatments (control or both snakes present in quadrant)*.

**Figure 3:**
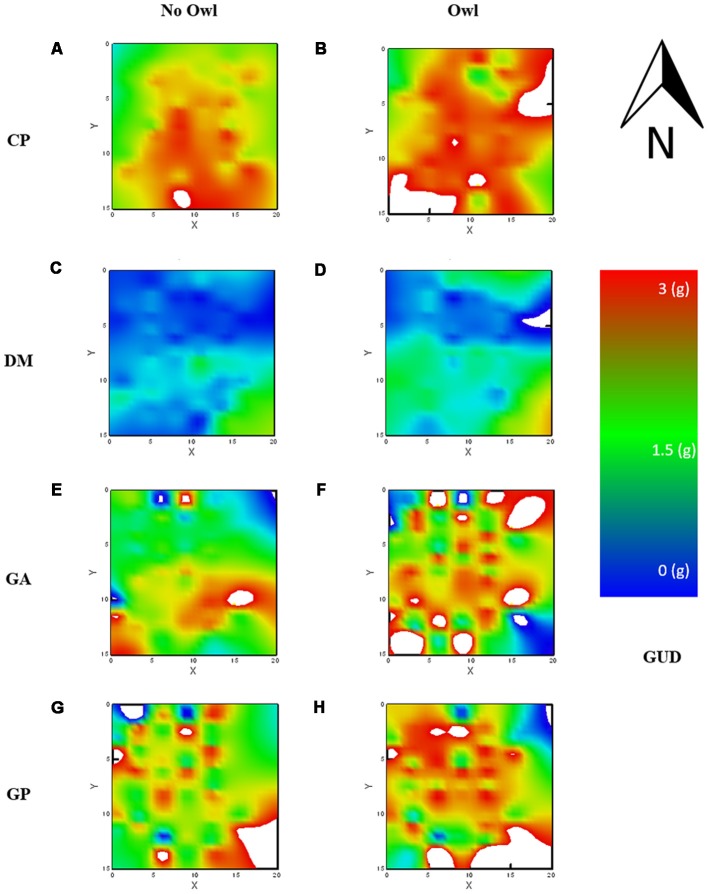
Landscape of fear (LOF) charted using GUDs and using a distance, weighted least squares (DWLS) smoothing function to generate the raster. Warm colors [red (3 g)—yellow (2 g)] reflect perceived danger (high GUDs) and cold colors [blue (0 g)—green (1 g)] relative safety (low GUDs). Each chart is identified by its location along species rows and columns of without and with owl presence, for CP (**A,B**) respectively, for DM (**C,D**) respectively, for GA (**E,F**), respectively, and for GP (**G,H**) respectively. *Dipodomys merriami* (DM) showed relatively weak risk perception regardless of owl presence, *Chaetodipus penicillatus* (CP) showed the strongest risk perception regardless of owl presence, and both GA and GP showed stronger risk perception when owls were present.

The average GUD, or mean “elevation” of the LOF, differed significantly for each species, as can be seen by the main effect of species on GUDs. The mean landscape elevation was similar for both gerbil species, GA and GP, with mean GUDs of 2.02 ± 0.05 g and 2.29 ± 0.04 g, respectively (Figures 3E–H). The mean elevation for the LOF of the heteromyid rodents, CP and DM, were at opposite extremes; low for DM with a mean of 0.71 ± 0.02 g and high for CP with a mean GUD of 2.50 ± 0.02 g (Figures 3A–D).

Overall, the rodents showed concordance in their perception of risk within each quadrant (Table 2). The overall pattern of distribution of risk increased towards the barrier between the quadrants likely as a result of permeability of the risk from snakes moving along the hardware cloth fencing (Supplementary Material, Appendix II).

**Table 2:** Freidman’s tests of concordance by quadrant (or snake treatment).

Quadrant	*X_f_^2^*	*p*-Value	*W*
North (Control)	160.77	<0.001	3.65
South (Snakes)	164.79	<0.001	3.75

**Figure 4:**
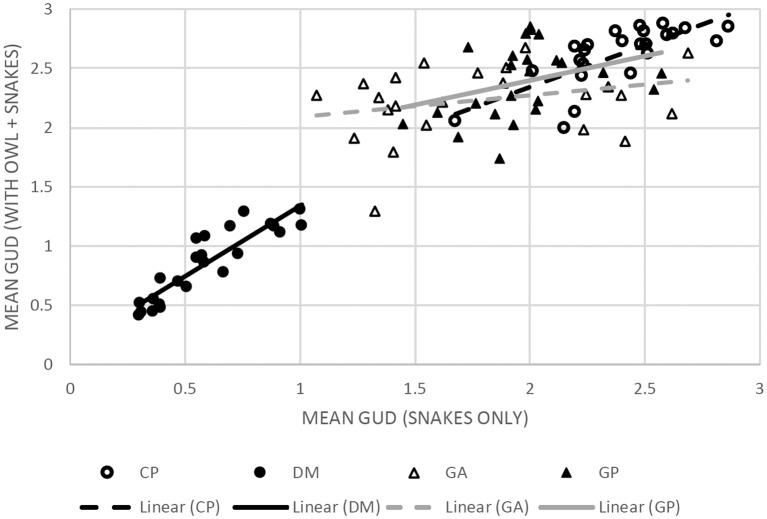
Scatter plots with linear regression lines for each of the four species correlating the mean GUD at each of the 24 stations as collected on nights with owls (*y*-axis) and without (*x*-axis). The (•) markers and black trendlines represent heteromyid rodents and (▲) and gray trendlines mark gerbilline species. The solid markers and solid trend lines represent the larger species (DM and GP, respectively) and the empty markers and dashed trend-lines represent the smaller species (CP and GA, respectively). The corresponding statistics are available in Table 2. The figure shows that when owls are present the level of risk perceived increases in the heteromyid rodents (CP, DM) while maintaining a relatively similar spatial distribution of risk. Meanwhile, the wide dispersions of values and minimal slope in the gerbils suggest that the shape of the LOFs of each species varies in risk distribution under each of the predation conditions.

To determine whether the LOF of the different species rises or falls with the risk of owl predation, we ran a regression analysis of mean GUD at a station when owls were present vs. when owls were absent. A positive slope with a tight correlation around the regression would show that the overall ranking of stations remains the same with and without owls; i.e., the LOF retains its shape with owls (Table 3, Figure 4). The heteromyids (kangaroo rat and pocket mouse) showed an increase in elevation of the LOF with owls and a tight relationship, suggesting little change in the topography of their respective LOFs. In contrast, the regressions for the two gerbils were non-significant. So while points on the landscape tended to rise with owls (a positive slope), these changes were not consistent (very low *R*^2^) as stations on the landscape perceived as safer without owls were often perceived as the most dangerous with owls.

**Table 3:** Regression analyses for each species as a function of the GUD per station with and without an owl effect.

Species	Linear regression equation	*R*^2^
*C. penicillatus*	y = 0.7046x + 0.9331	0.547
*D. merriami*	y = 1.1887x + 0.1518	0.807
*G. andersoni allenbyi*	y = 0.1825x + 1.9078	0.078
*G. pyramidum*	y = 0.412x + 1.574	0.117

## Discussion

This series of experiments applied the LOF, not as a theoretical model of spatial avoidance (Laundré et al., 2001), but as a measurable property of tradeoffs perceived by a population. The analyses we performed show that while the physical convergence is strong between the species, there appears to be a distinct pattern of divergence in the way heteromyids and gerbils comprehend the variations in risk based in the types of predators present and is generally distinct from the elements in the physical landscape.

We observed a degree of concordance between the four rodents which suggest that despite significant differences, all rodents perceived, or “understood,” the distribution of risk in the vivarium in a similar manner. We specifically found an abhorrence towards the central divider between the quadrants. The most striking of the observed differences was a clear differentiation between heteromyid and gerbilline rodents in the way the presence of the barn owl affected the shape and elevation of their LOFs.

Below, we compare and contrast the LOFs of the four species; discuss results with regards to rodent body size and taxonomic affiliation/continent of origin; and discuss the results with regards to the predator community.

(1) Did all species exhibit similar LOFs across the various conditions?

Landscape features may drive a common response in different species, as in the example of both elk and bison in Yellowstone avoiding waterways due to risk from wolf predation (Laundré et al., 2001). Features more likely to affect small mammal risk-perception may involve blocked sight lines (Embar et al., 2011) as in the case of gerbils in the same experimental vivarium and striped mice in South Africa (Abu Baker and Brown, 2010) avoiding habitat with thick vegetation cover. In these experiments, the rodents are clearly responding to the interaction of cues of predators as they interact with boundaries to movement, the partition between the quadrants. Not unlike the Yellowstone elk responding to the wolf risk by avoiding waterways, all rodents here avoid the fences when snake tracks and odors are present—suggestive of a combined effect of direct predatory cues and environmental information.

(2) Were similarities in the LOF most striking for species of similar body size, or ones originating from the same desert?

Our results did not show consistency between the patterns found in the LOFs of rodents within the same size classes nor within a family. Some patterns were replicated in different species, likely a result of similarities in behavioral traits.

(A) Size classes

In the small size class (GA and CP), the two species exhibited vastly different LOFs predominantly as a result of the height of the landscape (strongly evident in the random-forest). CP showed a high-elevation landscape (nearly unforaged), and the LOF for GA was of median magnitude (1/3 to half of the resources harvested). CP perceived the majority of the vivarium as risky, only willing to forage around the outer boundary of the vivarium. In contrast, the GA LOF was relatively “flat” and moderately safe in elevation (GUDs ~2 g) and gradually rose towards risky “peaks” (see Laundré et al., 2010), or islands (see Abu Baker and Brown, 2010), in the landscape (Figure 1B). Presumably, this is a consequence of the trellises where snakes were ambushing and open microhabitat patches where they were most vulnerable to owls.

Why do the effects of the risky features in the CP LOF attenuate so gradually across the entire landscape, while they are more restricted in the GA LOF? We believe the answers are attached to the specific adaptation of the pocket mice to climb into the branches of Creosote thereby escaping their predators (Rosenzweig, 1973). Our experiment, mimicking a bush with a branch covered trellis means the bushes do not offer escape paths in the way a natural occurring bush would when escaping snakes. If we were to put ourselves in the eye of a pocket mouse, this would mean that the only safe escape available to us is unreachable. Pocket mice, as opposed to kangaroo rats, do not have the powerful hind legs that allow them to jump out of harm’s way. With our trellis set-up designed to provide the maximum shelter from owls and foxes (see Embar et al., 2011) the similarity in GUDs under the bushes and in the open also suggests that the risk the pocket mice perceived from the snakes ambushing under the trellises and the risk from owls in the open was at least of equal value.

GAs, in comparison, take risks and forage under less favorable conditions. They forage on semi-stabilized dunes (Abramsky et al., 1990) and pick the “crumbs” left by stronger competitors (Kotler et al., 1993a). Both these behavioral patterns come at increased energetic and predation costs. To manage that risk they increase vigilance (Linder, 1988; Dall et al., 2001; Kotler et al., 2002). These behavioral adaptations result in an increase in GUDs across the landscape when risk is high, but also allow GAs to exploit more patches than CPs when risk is low. For CPs, their behavioral patterns observed in the wild suggest high selectivity towards safe habitats (Lemen and Rosenzweig, 1978; Brown et al., 1988) also reflected in a less diverse diet (Davidson et al., 1980). The CPs ability to enter torpor (Hayden and Lindberg, 1970) may also assist them in avoiding conflict and allows them to reduce movement in the environment when the conditions are not optimal. The relatively flat landscape suggests a limited dispersion of CPs even when the conditions were safer without the owls. These opposing strategies highlight the role of competition in these communities. The interplay of space, different anti-predator adaptations, and temporal use allow species in each community to co-exist with their close competitors. The LOF appears to support this pattern of behavior, a flat landscape when low risk from snakes is present, but turning to a spotty higher elevation (risky) map similar to GPs when the owls were flown in the vivarium.

The larger rodents exhibit substantially different LOFs both in their elevations and in “topographic” attributes. DM exhibit a flat landscape similar to its smaller family member, but as opposed to that of CP, that landscape is safe (low GUDs) and the safe areas are not centered on the edges. GPs showed a highly intricate weave of safe and risky patches, showing high sensitivity to variations in risk in the landscape (Figure 3).

With regard to its foraging patterns, GPs are regularly described as cream skimmers (Brown et al., 1997), meaning that they use their high harvest rates (small handling times) and low cost of changing patches (often due to fast locomotion) to move from patch to patch, discovering the richest patches sooner, and exploiting them when they are richest. This pattern was expressed beautifully in the LOF heat maps, which maintained an islands-of-fear pattern of localized risk even under low-risk conditions. This is when high harvest capacity within a short harvest time is most valuable. They move more, discover patches sooner, harvest them when they are richest, quit at high GUDs and move on in search of another rich patch especially at the time at which the environment is most dangerous (Dall et al., 2001; Kotler et al., 2002). The high sensitivity to risk, and where they are most likely to be ambushed, resulted in a LOF with a pattern similar to the one described by Abu Baker and Brown (2010) where striped mice avoid predation by genets in shrubs, creating a map of small islands of risk surrounded by safety zones.

In our system, when the level of risk increased in the environment (nights with an owl as well), GP altered the pattern of perceived risky zones, now predominantly the open microhabitat exposed to the owl and specific bushes fraught with high snake activity. This new landscape exhibits a focus on refuge identification as is best exemplified by the stark contrast between the LOF on nights when an owl was present and when the owl was not present (Figure 3).

The LOF of the kangaroo rats (DM) is flat and low, this suggests little fear. The management of predation risk in kangaroo rats (other *Dipodomys* species) is well documented and includes the following: high auditory acuity (Webster, 1961, 1962; Webster and Webster, 1971, 1979; Webster and Strother, 1972), foot drumming (Randall, 1997, 2001), kicking sand towards the predator (Bouskila, 1995), enlarged hind limbs (Biewener and Blickhan, 1988) and an ability to select safe habitats (Brown, 1988; Randall and Boltas King, 2001). Combined, these provide for a tremendous escape ability (Whitford et al., 2017). It is in this context (low risk) that the entire landscape slightly rises in response to the owl; overall, a less “dramatic” change than for the three other species.

(b) Intra-Continental Comparison

As indicated previously, the patterns of the four species were quite different from each other. Thus, even within the same rodent lineages (gerbilline and heteromyid) the way by which risk is assessed may not be inherited from their common ancestor, it appears to be more plastic and species-specific. Still, some general similarities exist within families. The plasticity in both gerbil species’ LOFs suggests the effects of predators was not cumulative. Heteromyids, on the other hand, exhibited fixed-shape landscapes that rose and fell based on the intensity of risk of predation.

Using a macroevolutionary lens, these behavioral patterns offer a glimpse into the evolution of desert systems on both continents. The behavioral similarities in the response of the gerbils, i.e., the reorganization of the LOF, suggests the important role that owls took in shaping the granivorous rodent community through sensitivity to those predator cues (Bleicher, 2012; Bleicher et al., 2018b), foraging strategies (Kotler et al., 1993a; Embar et al., 2011) and temporal variability (Kotler et al., 1993b). When owls were present, they take the brunt of the attention.

In contrast, the heteromyid rodents decreased foraging slightly on the nights when predation risk by owls was added, even in the areas with snakes absent (Figure 3). We hypothesize that sharing an evolutionary history with snakes that have heat-sensing ability drives a baseline of risk on which any combination of predators has a cumulative impact and demands the basic anti-predator awareness. Thirteen species of rattlesnakes call the Great Basin deserts home, and all of these possess the infra-red sensory ability (Fowlie, 1965). In comparison, there are only five vipers in the Negev and the Sahara, and they all are limited in their hunting on moonless nights (Joger, 1984). The high diversity of lethal predators in North America suggests the pressure to manage the risk from snakes has been a lot more important in the evolution of heteromyids. From that importance stems their sensitivity and acuity to the presence and activity patterns of the snakes they encounter. This also suggests that the adaptations, both physical and behavioral, for managing the risk from snakes would be a lot more extreme, but benefit evasion from all predators (see Webster, 1962). With the species we studied, the kangaroo rats are able to take the risk due to the number of physical adaptations they have to manage the risk (as mentioned before) but none as effective as bipedal locomotion—allowing for reverse locomotion (Randall, 2001). Pocket mice, on the other hand, use a combination of habitat selection (dense vegetation) and when conditions are not favorable they have been observed to enter a torpor state to mitigate the energy loss resulting in limited foraging (Hayden and Lindberg, 1970).

(3) Did owls cause changes only in the elevation of the LOF or in its shape as well?

The answer to this question is not as straight forward as the analysis would suggest. As stated previously, the patterns of behavior are not consistent between species of the same size class, nor between species sharing evolutionary history within the same desert system. The spatial changes in risk in gerbilline species based on the predator community suggest completely new LOFs. The change in heteromyid LOFs predominantly exhibited an elevation rise; however, the rise only occurred at the risky stations, causing the landscape to fold and increase in rugosity. From the four examples this research provides, we can only draw a general conclusion—the species-specific adaptations characterize the manner in which the LOF changes in response to varying risk conditions. To conclude, spatial patterns of anti-predator behaviors are a useful tool to compare species response in manipulative experiments. They reveal behavioral patterns that address the assessment of risk and its interaction with environmental attributes in the landscape. These behavioral patterns provide insight into driving forces (ecological and macroevolutionary) that explain the interactions between (and within) trophic levels.

We have demonstrated that the accumulation of predation risk from multiple predators can change the LOF, the way species assess risk in their environment, in one of two major fashions: (1) A LOF will change in topographical features, specifically in species that assess the risk in the environment based on the highest risk factor as was exemplified by the gerbil species; and (2) a LOF can rise and fall, specifically in species where the response to predators is spatially fixed and risk is cumulative.

## Data Availability

All datasets generated for this study are included in the manuscript and/or the Supplementary Files.

## Ethics Statement

The permits for this project were obtained from Ben Gurion University of the Negev Ethics in Animal Research Committee (Permit IL-73-11-2009) and animal shipping, handling and experimentation permits from the Israel Nature and National Parks Authority (INPA) permits (2011/38131 and 2012/12524). This is a publication 1015 of the Mitrani Department for Desert Ecology.

## Author Contributions

The data collection, analysis and main drafting of the manuscript was preformed by SB. JB and BK filled advisory rolls, were significantly involved in the experimental design, edited the manuscript and were responsible for the funding.

## Conflict of Interest Statement

The authors declare that the research was conducted in the absence of any commercial or financial relationships that could be construed as a potential conflict of interest.
