# Supplementary material for: Comparing Plasticity of Response to Perceived Risk in the Textbook Example of Convergent Evolution of Desert Rodents and Their Predators; a Manipulative Study Employing the Landscape of Fear
Source: Front Behav Neurosci. 2019 Mar 22;13:58. doi: 10.3389/fnbeh.2019.00058 (PMC6440367; doi:10.3389/fnbeh.2019.00058)
Supplement: Supplementary file 1 [file Data_Sheet_1.PDF]

Appendix I. Table of Tree Structure for Random-Forest Analysis

Child Node 1	Child Node 2	Node Size (N)	Node Mean	Node Variance	Split Variable	Split Category	Split Category
2	3	938	1.86	1.08	Species	DM	
4	5	257	0.69	0.29	Owl	No-Owl	
6	7	123	0.62	0.29	Snakes	No-Snakes	
		49	0.36	0.13			
8	9	74	0.79	0.33	Microhabitat	Bush	
		41	0.74	0.19			
		33	0.85	0.5			
10	11	134	0.76	0.28	Snakes	No-Snakes	
		57	0.55	0.17			
12	13	77	0.91	0.3	Microhabitat	Open	
		26	0.71	0.34			
		51	1.01	0.25			
14	15	681	2.31	0.66	Species	GA	GP
16	17	390	2.15	0.8	Microhabitat	Bush	
18	19	206	2.06	0.85	Owl	No-Owl	
20	21	113	1.88	0.8	Species	GA	
		34	1.82	0.91			
22	23	79	1.91	0.75	Snakes	No-Snakes	
		33	1.84	0.58			
		46	1.95	0.86			
24	25	93	2.26	0.82	Snakes	No-Snakes	
		50	2.23	0.91			
		43	2.31	0.72			
26	27	184	2.26	0.72	Species	GA	
28	29	75	2.08	0.83	Owl	No-Owl	
		30	1.77	0.92			
		45	2.29	0.67			
30	31	109	2.38	0.6	Owl	No-Owl	
		59	2.15	0.68			
		50	2.66	0.36			
32	33	291	2.51	0.41	Snakes	No-Snakes	
34	35	150	2.3	0.48	Microhabitat	Bush	
		75	2.19	0.54			
		75	2.41	0.39			
36	37	141	2.74	0.25	Owl	No-Owl	
38	39	70	2.68	0.3	Microhabitat	Bush	
		34	2.63	0.33			
		36	2.72	0.26			
40	41	71	2.79	0.2	Microhabitat	Bush	
		40	2.75	0.19			
		31	2.85	0.22			
