# Supplementary material for: Comparing Plasticity of Response to Perceived Risk in the Textbook Example of Convergent Evolution of Desert Rodents and Their Predators; a Manipulative Study Employing the Landscape of Fear
Source: Front Behav Neurosci. 2019 Mar 22;13:58. doi: 10.3389/fnbeh.2019.00058 (PMC6440367; doi:10.3389/fnbeh.2019.00058)

APPENDIX II – distribution of risk (mean rank from 1 (low)-12(high) risk) based on mean rank (between all species). The trendline (polynomial 2nd order) shows the increase in risk the species experience towards the central divider, a see-through hardware cloth fence.

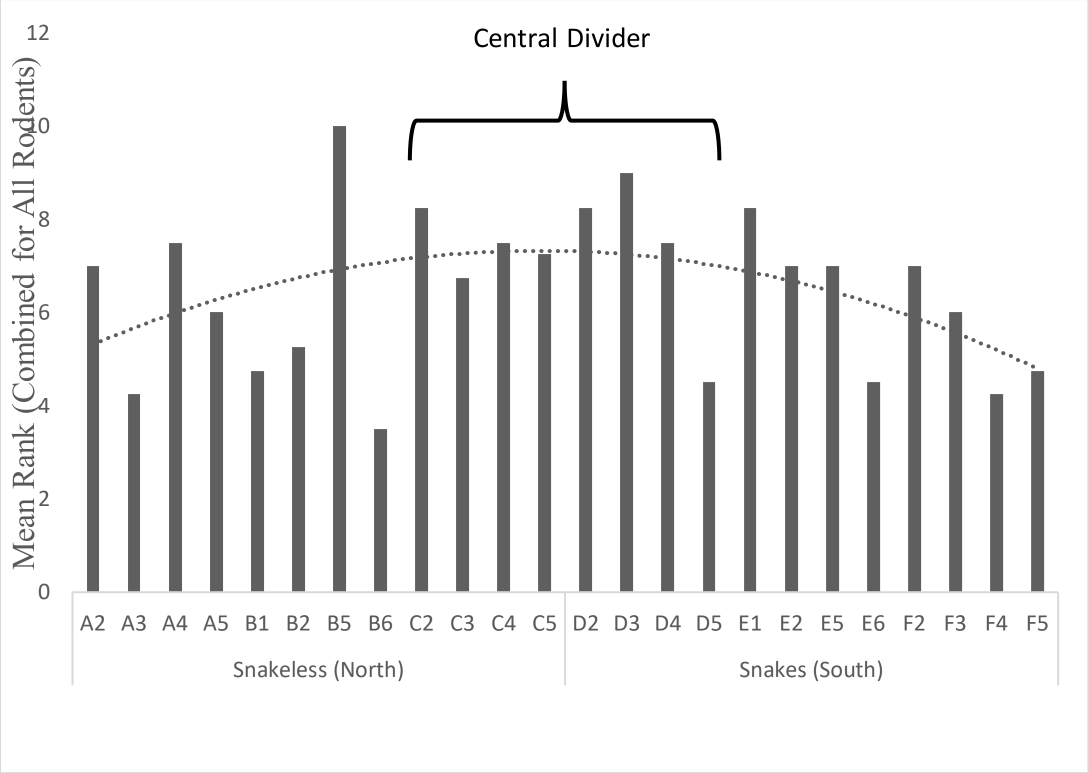

Supplement: Supplementary file 2 [file Data_Sheet_2.PDF]
